# Comprehensive security, disinformation, and COVID-19: An analysis of the impacts of mis- and disinformation and populist narratives during the pandemic

**DOI:** 10.12688/openreseurope.16733.1

**Published:** 2023-11-23

**Authors:** Arsalan Bilal, Gunhild Hoogensen Gjørv, Marc Lanteigne, Rachele Brancaleoni, Jardar Gjørv, Daniele Gui, Justyna Karolina Kielar, Caleb Aluola, Sabina Magalini

**Affiliations:** 1 Centre for Peace Studies, UiT The Arctic University of Norway, Tromsø, Troms, N9037, Norway; 2 Department of Social Sciences, UiT The Arctic University of Norway, Tromsø, Troms, N9037, Norway; 3 Fondazione Policlinico Universitario A. Gemelli IRCCS, Rome, Italy; 4 Universita Cattolica del Sacro Cuore, Milan, Lombardy, Italy

**Keywords:** Security, Comprehensive Security, Disinformation, Covid-19, Pandemic, Populism

## Abstract

The COVID-19 pandemic has generated many fundamental and challenging implications regarding security, for both states and people. This article addresses the pandemic as a security threat, whereby societal and human dimensions of security are intertwined with the narrower (so-called traditional) state dimensions, culminating in comprehensive security. This article uses mixed methods, combining desk research and a selection of narratives or stories from several parts of the world that signify how the intersection of disinformation and populist discourses exacerbated the COVID-19 security challenges. These are analysed through an innovative comprehensive security analytical approach. Drawing on both security theory and policy, the article examines how the COVID-19 pandemic jeopardised security on multiple levels. First, the state’s capacity to effectively act and deliver in the domestic sphere waned. Second, the social contract between the state and its citizens eroded as public trust dissipated. This article argues, however, that the most pervasive threat to security during the pandemic pertained to the exploitation of the information domain in relation to the state, society, and people. The article interrogates how mis- and disinformation about the pandemic compounded and exacerbated the security challenges it posed, often relying on existing narratives within right-wing populism movements to increase mistrust and discontent. These largely right-wing populist narratives contributed to broadening the gap between states and people, besides weakening public compliance with state health security measures. The nature of populism and the narratives of particularly right-wing populism contributed to increases in fragmentation, polarisation, and discrimination impacting societal trust. The article concludes with recommendations to mitigate the adverse impacts of mis- and disinformation, including reinvigorating the relationship between state institutions and the people to strengthen comprehensive security.

## Introduction

The COVID-19 virus spread rapidly after being first reported in the Chinese city of Wuhan in December 2019. ^1^ Within three months, COVID-19 was declared a pandemic with several regions of the world, including Europe, were affected. ^2^ Despite having been aware that a future pandemic was likely, most states were still caught out as being poorly prepared. By the end of March 2020, over 800,000 had been infected with over 40,000 dead. ^3^ The crisis upended daily life for the majority of people around the world, and severely impacted the global economy. ^4^ By December 2022, the official global death toll from COVID-19 was over 6.6 million. ^5^ Other estimates suggest the actual death rate has been even higher. ^6^ For example, the World Health Organization (WHO) has also claimed there were around fifteen million excess deaths associated with COVID-19. ^7^ Compounding the rate of infection was the fact that the development, distribution, and administration of vaccines to fight the virus was regionally and globally uneven. ^8^

The United Nations Secretary General deemed COVID-19 “the most challenging crisis” the world had faced since the Second World War. ^9^ Following the outbreak, governments scrambled to develop and enforce measures intended to mitigate the health, socio-political, and economic challenges that the pandemic posed. Through these measures, government authorities attempted to change and regulate their citizens’ behaviour in ways that were unheard of or unsettling for many. Most people, regardless of government type (democratic or authoritarian) were not accustomed to the state compelling them to stay indoors, shutter businesses, maintain social distance, avoid inter-household mixing, and so on. Public compliance with these measures, which were either arbitrarily or forcefully (or both) implemented, remained a major issue for authorities to grapple with. ^10^ The challenges created by the pandemic itself were compounded by mis- and disinformation, which intersected with populism, exacerbating issues of trust and public compliance in a crisis. These developments raise key questions regarding important links between state and individual/human security, how states conceive citizens’ security, and how people view their own security vis-à-vis the state. The COVID-19 pandemic was more than just a colossal health challenge: it directly and indirectly impinged on and threatened state, societal, and individual security in several ways.

Employing a comprehensive security analysis, we ask in what ways can, and/or does, the intersection of COVID-19 disinformation and populist narratives adversely affect national, societal and human security?

## Analytical approach and methods

We employ a multi-actor, multi-sector security analytical approach, rooted in theories of security, that recognizes and accounts for the complexities of today’s security environment. ^11^ The actors that declare or articulate a security threat can be state or non-state based, including policymakers, ministries and militaries representing state security perceptions, but also security actors representing local or community members, religious and or cultural groups, opposition groups (can be armed periodically), media, business and industry, non-governmental agencies (humanitarian, environmental, etc), and research. As mentioned above, and inspired by the domains identified in recent concept developments around hybrid threats, ^12^ we expand on Buzan, Wæver and de Wilde’s sectors, including military, political, economic, societal and environmental but adding climate, energy, human (health, well-being, freedoms, etc.), information, cyber, and space. ^13^ These sectors of security reflect values (as categories) and threat trajectories that can be operationalised on multiple levels (individual/human, societal/subnational, state/national, regional, international/global). We thus understand security as value-based, context-contingent, and a process (if not product) of actors employing various practices or means to protect the values most central to their perception of survival over time. One or more sectors can be applicable dependent upon the context under examination.

For the purposes of this analysis, we want to highlight the interactions between political/state and society as well as cyber, information and human security sectors. Political security revolves around the organisational strength of states as well as the governmental structures, institutions, and ideologies that furnish them with legitimacy. Political security is often tightly connected to military security, where the external security of the state is dependent upon the use of military force. ^14^ However, a solely state (military and political) based security lens misses out on how security and insecurity operate on other levels and/or in conjunction with other sectors. In the case of examining the security threats of both a pandemic and the vital information needed to provide increased security in the face of a pandemic, we need to increase our focus on the societal, human, cyber, and information sectors of security.

With respect to the empirical data collected to draw generalisations, we engaged in desk research drawing from academic and policy research content from relevant national and international organisations including the WHO and EU, as well as secondary sources of information. These included scholarly books and journal articles, policy reports, as well as website material, magazine content, and newspaper articles. The data was collected qualitatively whereby researchers utilised their expert knowledge in combination with the narrative data obtained through the above diverse sources, to produce a sample that was relevant for the purpose of the study. Since the process is marked by a certain degree of subjectivity and remains contingent upon the expertise and experience of the researchers undertaking the study, another group of experts can potentially arrive at a different sample of content if the research is replicated. ^15^ However, rather than contradicting the work conducted in this study, this approach contributes to an increasing accumulation of relevant analysis and knowledge that increases our overall capacity to understand the security dynamics arising in the interactions between forms of populism and practices of disinformation during times of crisis. Such an approach reflects the interpretative nature of such studies whereby subjective meanings of the social and political world are emphasised. ^16^

The empirical data we analysed was assessed in accordance to the chosen theoretical approach developed in our own previous scholarly work combined with academic contributions from primarily, but not exclusively, security studies scholarship. For the purpose of meticulously analysing both empirical data in relation to our theoretical parameters, we employed narrative analysis which allows researchers to qualitatively connect events in a meaningful way. ^17^ Narrative analysis entails finding out how meaning is implicit in the sequence of events as well as the surrounding circumstances. Such understanding is instrumental in dissecting causal links and correlations between events and surmising critical conclusions out of them in relation to the research questions. ^18^

This research follows the thematic model of narrative analysis. To this end, we rely on a series of stories (narrative data) from around the world to offer meaningful explanations that pertain to the research problem and research questions. By analysing and interpreting meaningful parts of narratives that we put under scrutiny, we develop an approach that helps us identify and explicate thematic elements flowing from discourses marked by a sequential order. ^19^ We use these thematic elements to reflect upon the conceptual and theoretical propositions put forth in this study regarding comprehensive security and disinformation around the COVID-19 crisis. Our conceptual/theoretical and methodological models thus remain intertwined.

## Effects of COVID-19 on security and the information realm

While COVID-19 progressively claimed millions of lives globally, states were trying to regulate public behaviour through unprecedented complete and partial lockdowns. ^20^ The pandemic, and the collective responses, had adverse implications for global, regional, and national economies. ^21^ As a United States (US) Congressional Research Service report highlighted, it “affected the $90 trillion global economy beyond anything experienced in nearly a century.” ^22^ The pandemic caused the worst economic crisis since the 1930s. ^23^ States around the world were hit by recession, and the global economy contracted by 3.4% in 2020.

Developing countries, their emerging markets, and their people will most likely bear the brunt of the pandemic in the longer term. The world experienced widespread unemployment, and COVID-19 disrupted global efforts to mitigate abject poverty. ^24^ The income losses and other compounding factors set poverty reduction back by an average of four years. By 2030, 588 million people around the world could still be living in extreme poverty—fifty million more than what was forecast prior to the breakout of COVID-19. ^25^ The pandemic also deepened other health challenges, especially in the developing countries, where healthcare systems were stretched beyond their limits. As a result, deaths from diseases including tuberculosis and malaria rose. ^26^

The question of whether the COVID-19 pandemic posed a security challenge *per se* is wrapped up in what security is and whose security matters. If something is a security challenge because a state’s survival, power, capacity, and influence are undercut, then that would rely upon a narrower, realist definition. ^27^ If something is a security challenge because people’s survival and well-being are undermined, then that would mean employing a wider understanding of security, including human security. ^28^ If the state’s and people’s security are complementary, or mutually constituted, then it might be possible to balance the two so they reinforce and support each other – a form of comprehensive security. ^29^

The COVID-19 pandemic jeopardised security on the levels of the state, society, and people. First, the state’s capacity to effectively and decisively act in the domestic sphere waned to some degree amid widespread economic and socio-political instability. Second, the social contract between the state and its citizens eroded. Several countries experienced unrest—especially once the initial apocalyptic fear tapered off—with the people feeling aggrieved or marginalised and calling upon their governments to perform their basic functions and provide public services. ^30^ Third, as we earlier highlighted, the lethality, spread, and adverse economic implications of the virus imperilled people’s survival, livelihood, and dignity. People found themselves embroiled in a precarious situation and bore the brunt of the suffering. They were contracting, and significant numbers dying, of the virus, their basic rights and freedoms were infringed, and their prospects to afford necessities and live a decent life diminished. ^31^

In addition to these, some of the gravest and most worrisome repercussions of COVID-19 on security are the exploitation of the information sphere in relation to the state, society, and people. In the aftermath of the outbreak, a statement co-authored by thirteen countries and signed by 132 states signified there was a threat to security as dangerous as the pandemic itself: the *infodemic,* the misinformation, disinformation, and fake news pandemic. ^32^ This was termed a “dangerous” threat to “security.” ^33^ Mis- and disinformation about the pandemic fomented confusion and risk-taking behaviour (such as refusing vaccination and refusal to abide by lockdown or social-distancing regulations), both of which are major impediments to public health. The spread and amplification of harmful messages—especially on digital and social media—contributed to exacerbating and prolonging the pandemic. ^34^

The challenges arising during the pandemic put the notion of freedom of speech or expression, especially in times of crisis, under scrutiny. Freedom of expression is not only the bedrock of democratic pluralism but also remains essential for the realisation of basic human rights. Such freedom must extend beyond popular and uncontroversial ideas or worldviews, though of course it comes with certain caveats insofar as freedom of expression ought not to be a license for socio-political threats like hate speech. ^35^

The COVID-19 pandemic foregrounded the problem that freedom of expression could be exploited to undermine the security of not only certain people but also the broader society and state. In other words, one person’s freedom of expression can not only harm another person or group of people, but its impact, amplified by current technologies, can also harm society and even the state. During the COVID-19 crisis, mis- and disinformation became threats. We will discuss mis- and disinformation later in this article, but in a nutshell, they can emasculate the state’s capacity to assert its authority, undermine public trust in government institutions, stunt compliance with the latter, reduce confidence in scientific knowledge and bodies, and stoke divisions among people at the societal and political levels. Mis- and disinformation, particularly during crisis, are typical “under threshold” or non-military threats, that demand a reconceptualization of the security framework as for today’s complex world.

## Reconceptualising security to reflect current complexities

The COVID-19 pandemic, and the crises which emanated from it, have thrown a spotlight on what security means, and what it takes to be secure. Scholars have pointed out the shortcomings of narrower definitions found in traditional or national security, which revolves around the state and military strength. ^36^ The concept of security is so prevalent and relevant that it is analysed as a feature of everyday life. ^37^ At the same time, the meaning of “security” continues to be contested, meaning, there is no agreement on the definition. ^38^ Security has remained a derivative concept that people understand based on their political orientation, personal experience and philosophical worldview. These contrasting understandings of security also render it difficult to define what is a threat, and to whom, and who has the power to decide. Moreover, intricate socio-political processes generate different interpretations of current events, interpretations that are based on perceptions or misperceptions. ^39^

This line of reasoning raises a set of questions that pertain to security and threats today. What is the nature of the threat at hand? Who or what is the source of the threats? How is the entity seeking freedom from the identified threats? Is the referent object of security an individual, group, nation, state, or all of these? Provided that all these questions are answered, can security be optimally achieved for all the relevant actors involved, and if so, how? ^40^

The state has long dominated the discussion of security, often claimed as the only significant security actor. ^41^ The *state* has three elements: the overarching idea or purpose of its existence; the institutions defined by government machinery (the legislative, administrative and judicial organs), coupled with the norms, laws and procedures that underpin them; and the physical base of its population and territory. ^42^ We hold that the state remains interwoven with the nonstate domains rather than remaining in absolute isolation. ^43^ In other words, the state cannot be detached from its ideational, institutional, physical, and social foundations. This argument indicates that a threat to any of these foundations shall be akin to a threat to the state. In the absence of societal security, there will be little political security because it is paramount to develop and maintain a sense of shared identity among the people under the larger umbrella of a nation-state. This nexus between the society and the state cements political security in a country insofar as the institutions have not only legitimacy but also, by the same token, the capacity to perform their functions.

The foregoing highlights that security—as well as threats—ought to be defined in a comprehensive manner. Security must go beyond the traditional realist paradigm that deems the state—considered a monolithic entity or power ^44^—as the actor that provides or ensures security and is to be secured against a multitude of threats. Furthermore, nonmilitary threats need to be taken into consideration regarding security. ^45^ Barry Buzan, and later Buzan, Wæver and de Wilde, argued for a broader framework of security that takes into account five security sectors that remain closely intertwined: military, political, economic, societal, and environmental threats. ^46^ The advancements made in the conceptualisation of hybrid threats has required an expansion of these sectors.

## Societal and human security in the cyber and information age

In the light of the notion that security is a multifaceted concept, the state cannot be regarded as the primary referent of security threats. In the contemporary era, society may be an equally important referent within security. In the 1990s, Buzan, Weaver and others concluded that ideological or traditional military security would become less relevant (within the European context) in the future. This highlighted the space for an alternate security agenda underpinned by societal security. They argued:

“Societal security concerns the ability of a society to persist in its essential character under changing conditions and possible or actual threats. More specifically, it is about the sustainability, within acceptable conditions for evolution, of traditional patterns of language, culture, association, and religious and national identity and custom. This definition makes it difficult to give any objective definition of when there is a threat to societal security… societal security is about situations where societies perceive a threat in identity terms.” ^47^

A society is essentially, as Giddens puts it, “a clustering of institutions combined with a feeling of common identity”. Institutions ought not to be understood literally – what rather defines a society is a high degree of inertia or generational continuity that is marked by a robust infrastructure of norms and values in the broad sense. ^48^ Societal security entails preserving a common identity that emanates from this inertia and continuity. It is also about the society’s ability to deal with issues of culture, language, shared history, etc. ^49^

In addition to the sector of societal security coupled with political security, a key approach or concept that goes beyond the traditional fixation on the state or military might be the sector of human security. This approach hinges on the notion that the security framework should be broadened in that the survival, well-being, and welfare of individuals are commensurate with the protection and power of the state. ^50^ It is thus a people-centric concept. Human security—which broadly entails freedom from fear and want—emerged from global policy debates in a Human Development Report developed by the United Nations Development Programme (UNDP) in 1994. It broadly subsumes seven categories: personal, political, food, health, economic, environment, and community security. ^51^

Human security has been criticised, primarily on the basis that the concept is too broadly defined and thus is difficult for policymakers to operationalise. While this might hold water to a certain extent, the approach has been instrumental in not deeming the state as the sole security referent, ^52^ the sole entity that is endangered and must be secured. ^53^ This does not mean that human security supplants state security—in fact, the state will be more secure if its citizens are secure, and they feel secure in the holistic sense. ^54^.

Human security entails comprehensively fortifying protection, equality, and empowerment of people in all spheres. ^55^ As the 1994 UNDP Human Development Report stresses, security—from the vantage point of human security—means protection from the constant threats of hunger, disease, crime, repression, and so on. Moreover, people need to be offered security so they are not subjected to unforeseen and detrimental disruptions to their daily lives at home, in the workplace, in communities, or in the environment. ^56^ Human security should not, however, be simply reduced to a question of adequate services and conditions provided by a state to its citizens. Human security is also a recognition of individual, civilian agency to define security and how individuals themselves seek and provide for their own security, based on their own, individual, security perceptions. It can therefore include the possibility of resistance, when state or societal security is perceived to threaten human or individual security. ^57^ The concept of *human security*, popularized by the 1994 UNDP Human Development Report, idealises equal power relations between actors, where the achievement of security is inclusive and representative. ^58^ There are instances however where *individual* security perspectives are not inclusive, but instead exclude “the other” or conceive of certain groups in society or the state as a threat to their own security, (for example, anti-migration, or anti-vaccination perspectives that target certain people or authorities as a threat). Ideally, human security can enhance societal security because people’s protection, well-being, equality, and empowerment translate into higher prospects of social inclusion and thus cohesion. Exclusionary individual security perceptions frequently align with exclusionary populist positions that pit “the pure people” against “the other.” ^59^

More recently the sectors of cyber and information have played increasingly relevant roles, not least concerning the creation and propagation of mis/disinformation. Cyber security “refers to the security of the digital environment, which constantly interactions with operations in the physical environment” where “the desired end state in which the cyber domain is reliable and in which its functioning is ensured,” resulting in “the protection of data and systems connected to the Internet.” ^60^ Cyber security encompasses a broad scope, protecting both the information and communication technology but also the “person(s) using resources in a cyber environment and. . . the protection of any other assets, including those belonging to society in general, that have been exposed to risk because of vulnerabilities stemming from the use of ICT (Information and Communication Technology).” ^61^ Information security is often combined or conflated with cyber security, but is a targeted asset in its own right. Information security can be defined as “the preservation of the confidentiality, integrity and availability of information.” ^62^ Information can be digital, dependent upon technologies and systems in cyberspace for its dissemination and protection and thus covered by cyber security. However, information security also pertains to information disseminated by papers (written or printed) and orally (lecture, conversation), as well as by radio or film transmission that may not be dependent upon cyber technologies.

Both cyber and information security have social implications as either being compromised can impact the wellbeing of human beings and society as a whole. In other words, in increasingly many contexts, cyber and information security are deeply intertwined with societal and human and/or individual, security, which in turn have significant implications for the successful or unsuccessful trajectories within and for political and state security.

## Mis-/disinformation and pandemic in the age of populism

The security challenges COVID-19 posed were influenced and exacerbated by mis- and disinformation, which were not by-products of the pandemic *per se* but intersected with it. Disinformation and misinformation are not purely modern phenomena—they predate the contemporary era. Nevertheless, they have become increasingly significant amid the technological boom of recent decades. New technologies in general, and digital and social media in particular, proffer ample opportunities to create and amplify fabricated, concocted, or distorted content. Such content is often peddled by states or non-state actors like populist figures – both in an attempt to influence if not manipulate general audiences - and shared wittingly or unwittingly by ordinary people. ^63^ The employment and exploitation of mis- and disinformation by states, by political demagogues, or by anti-state activists, has blurred the line between what is factually incorrect and what is independently verifiable. ^64^ The *problématique* we address entails not only mis- and disinformation but also how populist politics, in particular, can exacerbate these threats. Before delving into this relationship, we need to define mis-, disinformation, and populism.

Disinformation and misinformation are often used interchangeably, but they are not quite the same thing. *Misinformation* simply denotes wrong information. *Disinformation* is wrong information created and spread with the political motive of misleading people and exacerbating their socio-political vulnerabilities. Disinformation is generally aimed at inflicting harm on a person, entity, or country. ^65^ It exploits sociopolitical or cultural fault lines in a particular setting to exacerbate polarisation, instil fear in people, or undercut their trust in the state. ^66^ Disinformation might encompass, *inter alia*, fabricated and manipulated audio, visual, and textual content—including deepfakes (falsified images), readfakes (falsified text), and decontextualised material. ^67^ Disinformation targets and distorts the credibility and integrity of information, thus reducing information security. As the dissemination of disinformation is often digital (though not always), it additionally compromises cyber security. 

It is often very difficult—if not impossible—to establish a clear distinction between misinformation and disinformation, particularly when disinformation is forwarded by people who lack that political motive—people who may well believe they are passing along true information that will help others. For this article, we thus do not operationalise mis- and disinformation differently in relation to the research problem we address.

We must also conceptualise populism, which has become a catch-all term in contemporary political debates. ^68^ Scholars define populism in several ways – nevertheless, we conceptualise it in terms of identity politics that plays out vertically and horizontally within a polity. Originally, populism involved a democratic struggle against unjust political and economic elites. The term has been increasingly defined pejoratively, but populism was not a negative political phenomenon from the outset. ^69^ In recent decades, we have witnessed the waning of inclusive, democratic populism, and instead note that right-wing populism has taken precedence in different parts of the world. ^70^ It is underpinned by an ideology which pits a homogenous group of people against not only the elite but also the “dangerous others.” The approach aligns with notions of exclusive, individual security and societal security, as opposed to a more inclusive human security approach, that is pitted against “the other”, and/or the state. It is posited that the purported nexus between the elite and those deemed the “others” deprives the sovereign people of their identity, rights, voice, and values. ^71^ Moreover, the mainstream media is also often deplored and linked with the elites and political establishment in the populist discourse. This creates space for the creation, amplification, and spread of mis- and disinformation, especially on digital and social media. ^72^

## Right-wing populism and the media

Populist narratives around the prevalence of the COVID-19 virus, its effects, and the benefits and/or dangers of vaccines have come from both left- and right wing variants of populism. ^73^ However, the more extreme opposition to mainstream information about the virus has tended to come largely from the right-wing. ^74^ The scepticism with which right-wing populists often view the mainstream media brings us closer to relationship between populism and mis-/disinformation. While right-wing populists remain wary of the mainstream media, they also often contend that their access to traditional news media is purposefully curtailed because of bias and even corruption on the part of media owners and journalists. Populist actors have thus been both early adopters of and adept in digital technologies and communication. ^75^ Also, some scholars believe that digital connectivity platforms, especially social media, are often compatible with populist communication—which is direct, unmediated, and bypasses traditional gatekeepers. ^76^

Right-wing populism has been known to foment distrust towards national institutions, including those in Europe, ^77^ thereby undermining their legitimacy as well as undermining public trust and compliance. In conjunction with this, mis- and disinformation have become very problematic amid declining public trust in the mainstream press or media sources, even in several Western or European countries. ^78^ For instance, in Italy, public trust in news outlets has been particularly low in recent years. This is no surprise, considering people’s beliefs that journalism is swayed by politics and the commercial interests of the elite. ^79^ Mis- and disinformation dovetail with populist narratives that—by themselves—reduce public trust in the mainstream media. ^80^ Nevertheless, people often do not know they are exposed to disinformation, and they are not able to decipher how it has a bearing on not only their cognition but also their attitudes and behaviour at the socio-political level. ^81^ Advanced technological resources—particularly artificial intelligence that can imitate human cognition—can be utilised as threat multipliers in disinformation campaigns aimed at undermining a polity. ^82^ This is not to insinuate that disinformation campaigns always involve hostile foreign entities. Certain citizens, and their leaders, within a country also perpetrate them in pursuit of their personal and political agendas or for ideological reasons. ^83^

Since populism intersects with and deepens mistrust towards the mainstream media, the information that it generates and disseminates loses credibility. The interaction underpins a process whereby the information landscape is adversely altered. In relation to COVID-19, as empirical evidence suggests, a large number of people in several countries had conspiracy beliefs as they believed that the information shared on the mainstream media was not reliable or trustworthy. We want to highlight here that mistrust towards institutions and the media can be deemed both an antecedent and consequence of conspiracy beliefs and theories that permeate the societal space and information sphere. ^84^

## Disinformation and COVID-19

Mis- and disinformation were threats in relation to COVID-19 insofar as they not only exacerbated the challenges posed by the pandemic but also—on a broader level—contributed to dividing societies, thereby making them more polarised, vulnerable, and thus insecure. The WHO had warned that false or misleading information about the pandemic could intensify or prolong the disease outbreak amid a lot of people being unsure about what they needed to do to stay healthy and protect the people around them. This transpired to be true – since the pandemic affected virtually everyone around the world, a variety of networks and individuals peddled mis- and disinformation about COVID-19 through fast-paced social media, online forums, as well as instant messaging platforms. Even high-profile populist leaders—who had access to solid scientific and medical advice—were sometimes complicit – this played a crucial role in amplifying the problem in different parts of the world. ^85^

Even when states started rolling out vaccines and embarked upon steps to compel people to get them in a bid to bring the pandemic under control, mis- and disinformation remained a major impediment. In conjunction with other types of mis- and disinformation, false and misleading information about vaccines was rampant. Such messaging—which was facilitated by online digital and social media platforms—was associated with vaccine hesitancy in different countries around the world. ^86^ This became particularly problematic since complete vaccination of the adult population was seen as the most viable solution to the pandemic.

Some research suggested that mis-/disinformation about the pandemic was associated with not only increasing the number of COVID-19 cases but also death rates. ^87^ Also, as we have already mentioned, governments around the world had to resort to extraordinary measures which required compliance on the part of the citizens. These measures were then fused with the all-important mass vaccination campaigns. ^88^ However, “medical populism”—a term that was used to describe the politicisation of health issues along populist lines— ^89^and mis-/disinformation impeded government strategies to mitigate and end the pandemic. Such mis- and disinformation that underpinned populist discourses around the pandemic revolved around, inter alia, some of the following themes: ^90^

- *Simplification*: Contentions involving common-sense-oriented solutions to problems which are intrinsically complex are made. Moreover, the gravity of the pandemic is downplayed, and quick, magical fixes are propounded. Liberty and economy are sometimes pitted against public health.
- *Dramatisation*: Information about the pandemic is exaggerated and distorted. It is equated with conspiracies, emergency, war, and even apocalypse.
- *Divisions*: The society is divided not only vertically but also horizontally. The image of “the people” is created and they are pitted against powerful elites—such as the pharmaceutical industry, supranational bodies, medical authorities—as well as the “dangerous others” like the migrants who are deemed responsible for the crisis.
- *Knowledge claims*: Without any scientific basis, false knowledge claims are made. These range from contentions about and politicisation of the origins of the virus, its spread and impact, to proposed cures and solutions as well as prognoses. The pandemic can be declared a complete hoax and mortality rates highly exaggerated.

A variety of examples that pertain to the foregoing themes can be given to shed light on COVID-19 mis- and disinformation, which often played out in relation to populism. Cases in point include: unfounded claims that the anti-malaria drug hydroxychloroquine, alcohol, or certain nutrients and vitamins could cure COVID-19, facemasks were unnecessary, the virus originated in a Wuhan lab or a US lab in Ukraine, the lockdowns were ineffective, the vaccines contained microchips, receiving vaccines could make you magnetic or even alter your DNA, the vaccines caused variants, and people belonging to certain identity communities were responsible for the spread of the virus. All these assertions were unsubstantiated but were still spread wittingly or unwittingly, especially in the digital domain. ^91^

The scale on which such false and misleading information might have affected and influenced common people can be ascertained from the fact that a special report on mis- and disinformation around the pandemic—developed by the Strategic Communications and Information Division (StratCom) of the European External Action Service (EEAS)—noted that tens of millions of people were exposed to misleading or malicious information and propaganda in the digital space just in Europe. Moreover, despite being occasionally flagged by fact-checking websites, such content continued to go viral. ^92^ On social media, it was often peddled by pages or people with millions of followers. ^93^ As we will later explain in this article, this made not only public trust but also compliance difficult to achieve.

An interesting facet of mis- and disinformation about the pandemic was that it was sometimes fuelled by foreign actors in the context of global power competition between states. In particular, Russia, as well as China, were believed to be fanning false and misleading information about COVID. This was aimed at undercutting public trust in public institutions in the Western world that were responsible for executing policies and strategies to curb the spread of the virus. Moreover, public trust in Western vaccines was targeted for ulterior motives. ^94^ The idea was to not only dominate the information sphere amid an ongoing global power competition, ^95^ but also emasculate the overall security of countries in the Western bloc—particularly by creating and deepening divisions between countries part thereof. ^96^

While there is no denying that both mis- and disinformation as well as right-wing populism are often aided and abetted by foreign actors, they are not inherently external phenomena. In contrast, they are often home-grown threats to the state, society and people as well as the relationship between the three. As we have explained earlier, the predicaments arising from these can be attributed to growing mistrust towards established centres of power within polities as well as the legacy media whose credibility has been jeopardised amid populist politics. Such vulnerabilities are always exploited by hostile foreign actors, exacerbating the complex security challenges that we face in today’s world.

## The security challenges and the antidotes

We have indicated that COVID-19 was not only a health challenge but also a predicament that adversely affected security, if that concept is redefined, reconceptualised, and re-operationalised so it can address the complex problems engulfing today’s world. Mis- and disinformation compounded those security challenges. Mis- and disinformation about COVID-19 created or exploited polarisations or divisions at the societal level, thereby creating conditions for the outbreak of violence, conflict, human rights violations, and atrocities. ^97^ Moreover, mis- and disinformation created and intensified public mistrust in states, government institutions, policymakers, experts, and the mainstream media. ^98^ The security pitfalls of the pandemic and mis- and disinformation about it need to be holistically seen and explicated in relation to each other. A comprehensive diagnosis of this is essential for effective antidotes to the problems at hand. We argue that the crises generated by COVID-19 in general and related mis- or disinformation in particular have adversely impinged upon security in three major ways.

First, the pandemic, exacerbated by mis/disinformation and populism, eroded important trajectories of trust, either between people and authorities, or between groups of people themselves. The pandemic broadened the gulf between state and society by eroding people’s trust in political institutions and leaders. ^99^ It is pertinent to mention here that states and governments bank on public compliance with new norms and rules in a crisis. Compliance is in part contingent upon the support and respect people show for government policies and strategies, especially in democracies. ^100^ All this remains a function of trust in the state institutions and government apparatus – something that eroded because of the pandemic.

The implications of mistrust go beyond the perpetuation of the pandemic insofar as states’ capacity to address other problems is hindered in the long term. ^101^ Such mistrust has been compounded by mis- and disinformation about COVID-19. People are not only believing fabricated and concocted information but also fear they have been misled by their authorities—which represent the state. ^102^ This undercuts the legitimacy of the state—while remaining the Leviathan and maintaining monopolistic control over the domestic sphere—which derives its legitimacy from the people. ^103^ Legitimacy is the cornerstone of political, societal and human security in a country. Declining public trust has been translated into defiance insofar as people refuse to comply with the writ of the state. Although there is evidence that fear of consequences during the COVID-19 outbreak ensured compliance even amid declining public trust in several countries—including European ones ^104^—the problem remains worrisome. Fear, in the age of democracy and freedom, cannot be a sustainable guarantor of the viability of a state or polity. In relation to the fusion of state, societal, and human security, combined with information and cyber security, the security of the state cannot be ensured through the fears of people.

Once the overwhelming fear of COVID-19 among the people became diluted to a certain extent, ensuring compliance with government measures became an uphill task for states. This has degenerated into people palpably resisting and confronting state authorities in various countries. For instance, in several European cities, as states institutions scrambled to implement strict rules to curb the spread of the virus, huge numbers of people took to the streets in protest. Not only were there protests, but the demonstrations also often escalated into clashes. ^105^ In Brussels, both the capital of Belgium and the location of the EU, thousands clashed with the police. In Luxembourg, protestors stormed a Christmas market that banned the entry of unvaccinated people. ^106^ In Germany, around 700,000 people took part in a series of protests—some of which entailed direct violence. ^107^ In the Netherlands, thousands of protestors defied state authorities to protest COVID restrictions, resulting in clashes and arrests. ^108^ Similarly, in Italy, countrywide protests often turned violent. ^109^ Although all of these protests, as well as those in other parts of the world, drew disparate people with different motivations, what united them was anger with respect to not only COVID-19 policies but also political institutions. This remains symptomatic of people’s disillusionment with modern democracy and the dividends it can offer to the masses. COVID-19 protests were often spearheaded and promoted by radical groups, including populist parties, that increasingly exhibit extremist tendencies. ^110^

The second security implication of COVID-19 mis-/disinformation relates to how it fomented radicalisation, hate, and discrimination at the societal level in various countries, reflected also, in part, through exclusionary, individual security perspectives that isolate “the other”. These vulnerabilities continue to jeopardise societal and an inclusive human security which—as explained earlier – are both crucial prerequisites for the smooth functioning of any state. If the people are not provided with an environment conducive to preservation of inclusive shared identities and common associations, stability at the societal level will be compromised and hard to garner. Eventually, societal insecurity leads to state security being jeopardised, as the latter goes beyond military power.

Regarding how COVID-19 mis- and disinformation divided societies, many people held that the virus originated from out-groups or those who were considered not to “belong” to an exclusive common identity - the “others.” For instance, in the US, COVID-19 was frequently attributed to China; ^111^ as a result, there was a surge in crimes against Chinese and other Asians, who experienced not only discrimination but also racist attacks. ^112^ Populist rhetoric from leaders like President Donald Trump fanned disinformation by calling COVID-19 the “Chinese virus”, which greatly exacerbated the challenge. Disinformation about COVID-19 dovetailed with populist or exclusionary discourses and narratives on the socio-political level. ^113^ Foreigners from certain parts of the world were viewed as carriers of the virus and thus discriminated against by government authorities and the people. ^114^ Even people who are normally considered native to a region or country, were labelled as others were targeted within certain societies. A case in point is the use of disinformation campaigns to attribute the coronavirus to the Muslim community in India and Sri Lanka. ^115^ Another pertinent example was from France, where a decontextualised video was shared tens of thousands of times in the digital space that showed some young men—purportedly with minority backgrounds—brawling at a supermarket. ^116^ The video went viral in March 2020, at a time when fears had just heightened due to COVID-19 and the resulting lockdowns, with the apparent intent to exacerbate prejudice and hatred against young, male, minorities causing disruptions during such a vulnerable time. The video was from a year earlier in May 2019, capturing a gang rivalry in a store, where no employees were harmed. Such distortion, manipulation, and fabrication of information intensified societal polarisations and political cleavages in several countries around the world, ^117^ thereby deepening identity-related fault lines and undercutting societal and political security in a complex manner.

The third security dimension of COVID-19 mis- and disinformation revolves around the way in which certain vulnerable segments of society in various countries were disproportionately used as subject matter for, or targeted by, false and misleading narratives. With respect to the virus itself, some racial and ethnic minority groups in several countries were found to be more susceptible to infection. ^118^ This happened in tandem with widespread discrimination against minority groups in certain countries, exacerbating indirect impacts of COVID-19 on vulnerable racial and ethnic segments of the society. For instance, in Spain, 8% of migrants lost their jobs, compared to only 3% for the dominant population. The same trend was observed in France and Switzerland. ^119^ The disproportionate suffering of certain segments of the society in turn exacerbated divisions therein, thereby undercutting societal security. As certain segments of society felt they were at a disadvantage, their faith in the state as a guardian of everyone’s interests and security was endangered. This was greatly compounded by mis- and disinformation that disproportionately targeted minority or vulnerable groups in numerous countries. For instance, in the U.S., a study revealed that non-White people were more likely to accept COVID-19 disinformation. ^120^ Exclusion—whether perceived or real—negatively impacts social harmony and cohesion, thereby jeopardising human and societal security.

Taking into consideration the complexity of the mis-/disinformation threats and the way they play out, a range or policy and strategic responses have been propounded by experts over the years. These include developing and augmenting news or media literacy among the people so they can critically reflect on what they read and watch before forming and voicing their opinions; creating synergy between governments, businesses, and consumers to promote ethical practices; and promoting high-quality journalism to offset false, misleading, and harmful narratives. A lot of emphasis is placed on strengthening online accountability and regulation, ^121^ especially amid warnings that social media is not only rife with mis-/disinformation but is—by the same token—also undermining democracy and political institutions. ^122^ All of these solutions, in one way or another, hinge on the idea that mis- and disinformation—especially on the internet—are exponentially spread and amplified. The evidence in this article regarding how particularly foreign actors and sources peddle mis- and disinformation in pursuit of their political interests lends credence to such notions, but one should not underestimate the extent to which such menaces are home-grown phenomena.

The foregoing arguments direct attention toward key questions about the most effective ways to keep mis- and disinformation at bay. Should efforts that are geared toward mitigating mis- and disinformation curb people’s freedom of expression? We earlier highlighted that ideas, opinions, and beliefs are protected under the right to freedom of expression—even if they are based on false information or are absurd and ludicrous in nature. Yet, against the backdrop of this right being exploited or abused to inflict harm on the state and society, should freedom of expression come with certain strings or caveats? These questions are extremely difficult to answer. Nevertheless, two factors must be borne in mind while pondering them. First, curbing the freedom of expression—including expression that can be deemed inappropriate and unjustifiable—might inadvertently contribute to polarising societies that are already deeply divided. This impingement, restriction, or limitation risks unwittingly broadening the gulf between the state and society and between different communities (individual, polarized perceptions of security), at the societal level. Second, as an EU report on disinformation underscores, mis- and disinformation are—to varying degrees—phenomena that have become increasingly grounded in societies, including in the European region. These might not constitute the problem per se but instead reflects symptoms of other socio-political diseases, especially those related to social, political, and economic divisions and tensions. ^123^ The solution might entail putting countries’ own houses in order rather than being fixated on how external actors play a role in creating and amplifying problems such as mis- and disinformation.

Moreover, the preponderant focus while exploring solutions should be on how the broadening gulf between the state and its people, coupled with societal polarisation, contributes to furnishing internal and external actors with the impetus or opportunity to spread and amplify mis- and disinformation. Otherwise, as the EU report stresses, fighting mis- and disinformation “would be like treating the symptoms while letting the disease worsen.” ^124^ The bottom line is that an effective antidote to mis- and disinformation ought to involve bridging the chasms between state and society as well as among the people. Any set of solutions that do not build confidence and trust will probably fall short of meeting the challenge in a way that ensures sustenance and prosperity of the state and society.

One of the key issues with regards to disinformation and trust is the role of the civilians—or the ordinary people. How people think and act, reflective of their individual agency, in response to disinformation remains paramount. Trust between the state and society as well as between the people remain contingent, inter alia, on civilian agency. Contemporary digital and social media platforms allow disinformation to adversely impinge on civilian agency insofar as the people’s trust in the state apparatus as well each other is undermined. This plays out to the detriment of both the state and the society. It is important to mention here that the modern state and society remain interwoven. A robust, democratic state not only draws legitimacy from the society but also hinges on the stability of societal structures. Likewise, a vibrant society thrives on a responsive state. Trust should also not be conflated with blind compliance either; a robust democracy requires critique, debate, and disagreement to constantly strengthen the political system to which citizens contribute. Citizens need to be able to trust that engagement, disagreement, contestation, and compromise across societal actors is supported and protected by the state and the communities within.

Disinformation undercuts trust in democratic processes, between the state and the society, thereby creating conditions for instability and even implosion of the two. Moreover, at the societal level, disinformation exacerbates polarisations which can erode core values of peaceful coexistence and harmony but also contestation and debate reflecting pluralism in democracies. It is thus imperative to build and preserve trust as a bulwark against threats posed by mis- and disinformation in the digital age. By enhancing democratic values and societal cohesion, trust ensures that the policies and strategies developed to surmount mis- and disinformation come to fruition.

Trust is not unidimensional or single layered – it exists on multiple spheres and levels. For example, governments need people to have confidence in state organs to ensure compliance with their decisions. State institutions in a lot of countries, including those in the Western world, are losing their credibility vis-à-vis the public. In the United States, trust that the government will act in the right manner either always or most of the time has gone down from 73% in 1958 to 20% in 2022. ^125^ Levels of trust across European state have also declined, even after reduction of restrictions and return to more regular routines following COVID-19. ^126^

It is not only trust in the state that remains crucial. What remains equally important is people trusting each other within a polity, and the state trusting the people. While it is very difficult to gauge such trust, populist waves in several parts of the world, including the West, are symptomatic of rising social and political polarisations in national communities. Mistrust at the societal level jeopardises social harmony as well as a community’s social and political fabric. This affects decision-making processes.

Fortifying trust is critical when it comes to building resilience in the face of not only crises like the COVID-19 pandemic but also their intersections with mis- and disinformation that can acutely destabilise states and societies. Trust is thus essential to offset threats to our security on all levels. Building trust requires sustained measures to enhance the bond between the state and the people through meaningful transparency, ownership, and inclusiveness. Moreover, harmony and pluralism must be enhanced to overcome social polarisations and societal vulnerabilities.

## Main findings and conclusion

The COVID-19 pandemic has had far-reaching implications for the world. It served to undercut trust, and more broadly security, on the state, societal, and individual levels. Apart from the grave health, political, and economic implications, it had dire consequences for the state in terms of its functional capacity and its relationship with the people, as well as for societal cohesion related to durable conditions for well-being, equality, and peaceful existence.

Moreover, the pandemic particularly exacerbated the vulnerabilities of certain racial and ethnic minority communities by spawning a sense of marginalisation and alienation among them, particularly in liberal democracies. This was compounded by mis- and disinformation that added to their plight and to socio-political polarisation. Mis-/disinformation seems to have disproportionately influenced some minority groups, and combined with often reduced or marginal social conditions, made them more susceptible to contracting the virus. It also allowed them to be deemed as “the other” and—in turn—discriminated against at the hands of government authorities, but even more so by people operating on exclusionary individual security perceptions. This can also be associated with right-wing populism, which creates and deepens societal polarisations.

On a more general level, mis- and disinformation led to declining public trust in governments and leaders, thereby not only turning compliance into a challenge—especially in the absence of fear of consequences which, in itself, is problematic in democracies—but also pitting the people against their own authorities in certain countries. Mis- and disinformation about COVID-19 have dovetailed with populist and exclusionary discourses and narratives. Right-wing populism has created, intersected with, and amplified false and misleading information about COVID-19 pandemic as well as strategies to bring it under control. External powers—particularly Russia and China ^127^ —fanned this to the detriment of democratic polities.

In this context, while the state, society and individuals must embark upon measures to ensure that information that reaches the people is factual, it is important to stress that the most effective antidote to mis- and disinformation is rebuilding and augmenting their trust in the state. To this end, states must fuse democratic norms, principles, and processes with good governance as well as with trust, pluralism, and harmony at the societal level. This includes greater protection in cyber security (that information and other services are not tampered with via cyberspace), and provisions for security of information that is credible and reliable, combined with awareness of how inequalities and prejudices operate in society and foment political cleavages and polarisation, followed by measures to reduce these toxic trajectories. Without these, modern-day menaces like mis- and disinformation, as well as populism, are likely to continue wreaking havoc, especially in times of crisis.

## Ethics and consent

Ethical approval and consent were not required.

## Data Availability

No data outside of the cited sources are associated with this article.
