# Supplementary material for: Comprehensive security, disinformation, and COVID-19: An analysis of the impacts of mis- and disinformation and populist narratives during the pandemic
Source: Open Res Eur. 2023 Nov 23;3:209. [Version 1] doi: 10.12688/openreseurope.16733.1 (PMC10956505; doi:10.12688/openreseurope.16733.1)
Supplement: Peer review history

# Reviewer report: Reviewer response for version 1

**DOI:** 10.21956/openreseurope.18073.r38604

**Published:** 2024-03-11

**Authors:** Rubén Rivas-de-Roca

**Affiliations:** 1 Department of Communication and Sociology, Universidad Rey Juan Carlos, Móstoles, Community of Madrid, Spain

1. The text deals with a very relevant and timely topic, analyzing the COVID-19 as a security threat in which the information plays a key role. The authors develop a multi-sector analytical approach by using mixed methods. On this matter, security is understood in a broad way based on security theory and policy. This literature review is strong, but it could be reinforced with some foundational contributions.

 2. The manuscript employs a narrative analysis to present qualitative data and engages in desk research from outstanding organizations, as well as the use of secondary data. This method may be helpful, but, as the authors acknowledge, it presents high levels of subjectivity. Therefore, the lack of replicability should lead to assess this content more as an academic essay than as an empirical article.

 3. Following the above, the text sometimes seems to address too many topics. I would recommend including a paragraph on the structure of the article, explaining how the contextual background is built. Providing a list of objectives or research questions would also facilitate the comprehension of the research.

 4. Beyond some few criticisms, the paper shows how the mis- and disinformation about COVID-19 impacted on security and trust. The pandemic not only eroded the capacity of the state, but also the social contract between governments and citizens. Besides that, the rise of misinformation and disinformation put the state’s authority into question.

 5. Security is here considered as a multifaceted concept. This approach furthers our understanding on the increasingly important roles of the sectors of cyber and information in this field, particularly creating and spreading mis/disinformation. Although the review of documents is huge, I would suggest adding some recent literature that strengthens the academic soundness of the paper. For instance, it is necessary to contextualize how 2016 (because of Brexit and the elections campaign in the US) was a key moment for disinformation and post-truth:

- Enli, G. (2017). Twitter as arena for the authentic out-sider: exploring the social media campaigns of Trump and Clinton in the 2016 US presidential election. *European Journal of Communication*, 32(1), 50-61.

The current version of the paper carried out a good review on disinformation and COVID-19, together with references on the special affinity between populism and social media (Gerbaudo, 2018). Nevertheless, it would be also interesting to reflect on the substitution of the normative public sphere (Habermas, 1962) by a dissonant and disrupted public sphere (Bennett and Pfetsch, 2018), which allows the proliferation of disinformation and distrust in elites:

- Bennett and Livingston (2018 ^1^). The disinformation order: Disruptive communication and the decline of democratic institutions. *European Journal of Communication*, *33*(2), 122-139.
- Bennett and Pfetsch (2018 ^2^). Rethinking Political Communication in a Time of Disrupted Public Spheres. *Journal of Communication*, *68*(2), 243-253.
- Habermas, J. (1962/1989) *The Structural Transformation of the Public Sphere: An Inquiry into a Category of a Bourgeois Society*, trans. T. Burger and F. Lawrence. Cambridge, MA: MIT Press.

6. As the last proposal, the conclusions should be reworked. The section of results is well structured, but the conclusions seem short and do not summarize the main findings. It is also required to develop a proper discussion that contributes to a wider scholarly debate on the implications of COVID-19. Following research questions or objectives, the results could be presented in a jointed way. However, it would be worthy to remind in short sentences the purpose to overlap better with the previous sections. In the same vein, another recommendation is to provide here future lines of research to foster the knowledge on the intersection between security and information.

 In my opinion, the manuscript is drafted in a balanced way, but it would benefit from a clearer structure and deeper literature review. The manuscript is rigorous, contributes to advance the knowledge in the field and does not present serious problems in its current version. In short, this article means a relevant contribution that addresses timely issues by applying a narrative way.

If applicable, is the statistical analysis and its interpretation appropriate?

Not applicable

Is the study design appropriate and is the work technically sound?

Partly

Is the work clearly and accurately presented and does it engage with the current literature?

Yes

Are the conclusions drawn adequately supported by the results?

Yes

Are sufficient details of methods and analysis provided to allow replication by others?

Partly

Are all the source data and materials underlying the results available?

Yes

Reviewer Expertise:

Disinformation, political communication

I confirm that I have read this submission and believe that I have an appropriate level of expertise to confirm that it is of an acceptable scientific standard, however I have significant reservations, as outlined above.

## References

[1] : The disinformation order: Disruptive communication and the decline of democratic institutions. *European Journal of Communication* .2018;33(2) : 10.1177/0267323118760317 122-139 10.1177/0267323118760317

[2] : Rethinking Political Communication in a Time of Disrupted Public Spheres. *Journal of Communication* .2018;68(2) : 10.1093/joc/jqx017 243-253 10.1093/joc/jqx017

# Reviewer report: Reviewer response for version 1

**DOI:** 10.21956/openreseurope.18073.r37033

**Published:** 2024-03-11

**Authors:** Trishana Ramluckan

**Affiliations:** 1 University of KwaZulu-Natal, Durban, South Africa

The paper is well structured, however, there are a number of concerns regarding the paper which are as follows:

1. WHO is being used as the main source of information. Is it realistic to generalise on the topic based on a one-sided approach? Where does researcher bias begin and/or end? The authors did not consider the possible side-effects of the vaccines which may have contributed to the information being disseminated - this is important.
2. The analysis conducted by the authors remains of concern, i.e., lacks statistical rigour by using narratives of other authors. It is advised to consider both sides for the purpose of accuracy.
3. Overall, a well written paper, but how do the authors propose the prevention or rather mitigation of a false narrative? Who is responsible, accountable and liable for the dissemination of false narratives i.e., attribution?
4. Recommendations need to be stronger together with a detailed discussion and conclusion.

If applicable, is the statistical analysis and its interpretation appropriate?

Partly

Is the study design appropriate and is the work technically sound?

No

Is the work clearly and accurately presented and does it engage with the current literature?

Partly

Are the conclusions drawn adequately supported by the results?

Partly

Are sufficient details of methods and analysis provided to allow replication by others?

Not applicable

Are all the source data and materials underlying the results available?

Partly

Reviewer Expertise:

IHL Governance, Cybersecurity, IT Law, Information Security

I confirm that I have read this submission and believe that I have an appropriate level of expertise to confirm that it is of an acceptable scientific standard, however I have significant reservations, as outlined above.

# Reviewer report: Reviewer response for version 1

**DOI:** 10.21956/openreseurope.18073.r36645

**Published:** 2024-01-02

**Authors:** David Wright

**Affiliations:** 1 Trilateral Research, London, England, UK

This article focuses on the impact of mis- and disinformation about COVID on security, democracy and trust in societal institutions.

 In doing so, it argues that security must be understood in a broad sense, including human security, which itself can be unpacked into its different aspects.

 The article argues that the political reaction to COVID was not just a health crisis, but also a security crisis and that mis- and disinformation intersected to deleterious effect in prolonging the crisis and eroding trust within society and with our institutions. Populist politicians added to the societal divisions.

 The claims and statements in the article are well backed up by copious footnotes.

 There is some good and well-timed discussion on the multi-faceted concept of security. The distinction between information security and cybersecurity is useful. Equally useful is the point that it is often difficult to distinguish between misinformation and disinformation.

 The themes prevalent in COVID disinformation identified by the authors are good too: Simplification, Dramatisation, Divisions, Knowledge Claims, which are a useful complement to the DISARM framework.

 The article mentions the European External Action Service which has adopted the DISARM framework for reporting disinformation. It mentions the EEAS report on how tens of millions of people were exposed to mis- and disinformation about the pandemic, with deadly consequences. And more than the needless deaths, disinformation led citizens to distrust their governments, their medical authorities, their fellow citizens. It was also a challenge to security in its broadest sense.

 The article concludes that “states must fuse democratic norms, principles, and processes with good governance as well as with trust, pluralism, and harmony at the societal level”. Cynics among us might ask how likely those prospects are. The authors finally conclude that “Without these, modern-day menaces like mis- and disinformation, as well as populism, are likely to continue wreaking havoc, especially in times of crisis.” So it seems like we must accept the continuing havoc and devise a multiplicity of approaches to deflate as much mis- and disinformation as possible. Initiatives such as creation of the EEAS could usefully be replicated at the Member State level. More political attention is needed to create non-partisan, independent bodies with adequate budgets to inform the public about what is factual and what is fake.

 A few minor criticisms:

 A few of the statements in the article appear like pronouncements from on high: “Fear, in the age of democracy and freedom, cannot be a sustainable guarantor of the viability of a state or polity.”

 Some of the syntax is unduly officious: “We hold that the state remains interwoven…” on p. 6 or a little wordy: “We want to highlight here that…” on p. 10 or wrong verb tense: “Any set of solutions that do not build…” on p. 15, but these are tiny points.

 Overall, this is a well-crafted, well-written, well-structured article that merits publication to a wider audience who can benefit from its insights.

If applicable, is the statistical analysis and its interpretation appropriate?

Not applicable

Is the study design appropriate and is the work technically sound?

Yes

Is the work clearly and accurately presented and does it engage with the current literature?

Yes

Are the conclusions drawn adequately supported by the results?

Yes

Are sufficient details of methods and analysis provided to allow replication by others?

Yes

Are all the source data and materials underlying the results available?

Yes

Reviewer Expertise:

cybersecurity, disinformation

I confirm that I have read this submission and believe that I have an appropriate level of expertise to confirm that it is of an acceptable scientific standard.
